# One-Year Outcomes After Everolimus-Eluting Stents Implantation in Ostial Lesions of Left Anterior Descending Coronary Arteries

**DOI:** 10.4021/cr295w

**Published:** 2014-01-02

**Authors:** Zahra Golmohamadi, Sepideh Sokhanvar, Naser Aslanabadi, Samad Ghaffari, Bahram Sohrabi

**Affiliations:** a Cardiovascular Research Center, Shaheed Madani Heart Hospital, Medical Science University, Tabriz, Iran; b Ayatollah Mosavi Cardiology Department, Medical Science University, Zanjan, Iran

## Abstract

**Background:**

In recent years, stents are increasingly used in variety of coronary lesions. Ostial lesion of left anterior descending coronary artery (LAD) however remains a challenge area because of the invariable involvement of distal left main coronary artery (LMCA). This study was designed to evaluate the clinical and angiographic outcomes of everolimus-eluting stent (EES) implantation for ostial LAD.

**Methods:**

EESs were implanted in 45 consecutive patients with ostial LAD stenoses. For complete lesion coverage, stent positing was extended into the distal LMCA in 6 patients (13.3%) with intermediated LMCA narrowing. We assess MACE during one-year follow-up.

**Results:**

In-hospital success rate was 100%; neither cardiac death nor stent thrombosis in our patients, but two patients had myocardial infarction in non-related coronary artery during follow-up. Two patients had angiographic restenosis and underwent TLR. The cumulative MACE-free survival rate was 95.6% at one year.

**Conclusion:**

EES was in ostial LAD lesions with complete lesion coverage achieving high procedural success rate and acceptable clinical outcomes during one-year follow-up period.

## Introduction

The introduction of percutaneous coronary intervention (PCI) with stent implant has substantially shifted the treatment of coronary artery disease. However ostial left anterior descending (LAD) coronary lesions are a challenge for interventional cardiologist because these lesions usually involve the distal left main (LM) coronary artery [1]. Balloon angioplasty of ostial LAD lesions has been associated with lower rates of initial success and higher rates of procedural complication and late restenosis compared with that of nonostial lesions [2-4]. For these reasons such patients are often referred for coronary artery bypass grafting (CABG). Although various stents are currently used to reduce restenosis, the impacts of everolimus-eluting stent (EES) on early and late clinical outcomes have not been clearly elucidated for the ostial LAD lesions.

The present study evaluated early and late (one year) clinical and angiographic outcomes of patients who were following of EES in LAD lesions in our institution.

## Methods and Materials

From February 2008 to June 2011, we evaluated 45 consecutive patients with ostial LAD lesions at Shaheed Madani heart center in Tabriz. All patients had been treated with EES (Xience, Abbott Vascular international BVBA, 1831 Diegem, Belgium). Angiographic inclusion criteria were the presence of a lesion > 50% diameter stenosis rising within 3 mm of LAD orifice [5]. The following criteria were used for exclusion: acute myocardial infarction within 48 h, left ventricular dysfunction (ejection fraction < 40%), previous bypass surgery, significant involvement (≥ 50% of diameter stenosis) of LM or left circumflex (LCX) ostium, heavily calcified lesions.

Patients were followed up for at least 12 months. Repeat angiographic evaluation was left to the discretion of the physician. Clinical follow-up was performed by out patient visit. Angiographic follow-up was recommended between 6 and 12 months or earlier if clinically indicated by symptoms or documented myocardial ischemia.

### Interventional technique

Two different stenting approaches were performed at the operator’s discretion: the bifurcation stenting from the LM across the LAD ostium into the diseased branch (distal LM-LAD stenting) or the stenting right at the ostium of the diseased branch (focal ostial LAD stenting). In the distal LM-LAD stenting, a bifurcation technique with provisional side branch stenting was used. Provisional stenting of LCX was performed only in case of residual dissection, significant plaque shift, or residual side branch angiographic narrowing ≥ 50%. Final kissing balloon was routinely performed in patients treated with distal LM-LAD stenting. A strategy of direct stenting was performed and predilatation with standard balloon angioplasty was performed in very tight lesions. Stent deployment was performed by high-pressure balloon inflation to achieve optimal stent apposition. The type of stent to be implanted was EES. Each patient was adequately informed of alternative surgical treatment and signed and informed consent. At the beginning of the procedure, the patient received heparin (100 U/kg) bolus with the goal to achieve at activated clotting time > 250 s. Glycoprotein IIb/IIIa inhibitors were administered at the operator’s discretion. All patients were prescribed lifelong treatment with aspirin 100 mg daily. A loading dose of 300 to 600 mg of clopidogrel was administered if patient was not already on clopidogrel. Then continued 75 mg bid for two weeks. Double antiplatelet therapy (aspirin 100 mg daily and clopidogrel 75 mg daily) was recommended for 12 months.

### Angiographic analysis

All coronary angiograms (CAG) were analyzed by two experienced angiographers. Using contrast-filled guiding catheter for calibration and an on-line quantitative coronary angiographic system (Siemens, 077-21108, Germany) reference vessel diameter, percent diameter stenosis were measured before and after the intervention and at the follow-up from least foreshorten view.

### Clinical and angiographic follow-up

All patients were evaluated clinically by office visit at 1, 3, 6 and 12 months. Angiographic follow-up was recommended between 6 and 12 months or earlier if clinically indicated by symptoms or documented myocardial ischemia. In hospital events including death, myocardial infarction and repeat revascularization of the target lesion were evaluated. The primary end points were angiographic evidence of restenosis (diameter stenosis > 50%) and secondary end points were occurrence of any major cardiac events (death, myocardial infarction, and target lesion and target vessel revascularization) during the follow-up period. Myocardial infarction was diagnosed when cardiac enzymes were elevated three-fold or greater with chest pain ≥ 30 min or with the appearance of new electrocardiographic changes. Non-Q wave myocardial infarction was diagnosed when there were increases in cardiac enzymes to 2 times with chest pain ≥ 30 min without new Q waves on electrocardiogram. Stent thrombosis was defined as an acute coronary syndrome with angiographic documentation of vessel occlusion or thrombosis within or adjacent to a previously successfully stented vessel or in the absence of angiographic confirmation, acute myocardial infarction in the distribution of the treated vessel or death from cardiac causes not clearly attributable to other causes. Target vessel revascularization (TVR) was defined as any reintervention that was performed on the treated vessel. Target lesion revascularization (TLR) was defined as surgical or percutaneous revascularization that was driven by significant luminal narrowing > 50% within the stent or within the 5 mm borders proximal and distal to the stent.

### Statistical analysis

Data are expressed as mean ± one SD for continuous variable and as frequencies (%) for categorical variable. Cumulative analysis of event-free survival was expressed by Kaplan-Meier survival curve. Statistical analysis was performed with SPSS 14.

## Results

### Baseline characteristics

The baseline clinical and angiographic characteristics are summarized in Table 1.

**Table 1:** Baseline Characteristics of the 45 Patients

Characteristics	Mean ± SD or n (%)
Age	57.44 ± 11.63
Male	35 (77.7)
Risk factors	
Systemic hypertension	15 (33.3)
Diabetes	20 (44.4)
Total serum cholesterol ≥ 200	16 (35.5)
Current smoker	17 (37.7)
Family history	6 (13.3)
Unstable angina	22 (48.8)
ST elevation myocardial infarction > 2 days	16 (35.5)
Non-Q wave myocardial infarction	2 (4.4)
Left ventricular ejection fraction	45.5 ± 12.9
Diseased vessels, n	
1	26 (57.7)
2	14 (31.1)
3	5 (11.1)
Coronary lesion characteristics	
Discrete < 10 mm	16 (35.5)
Tubular 10 - 20 mm	23 (51.1)
Diffuse > 20 mm	7 (15.5)
Preintervention TIMI grade	
I	5 (11.1)
II	10 (22.2)
III	30 (66.6)
Thrombus	1 (2.2)

Forty-five patients (35 males and 10 females) with mean age of 57.4 ± 11.6 years were included in this study. The majority of these patients (22 patients 48.8%) had unstable angina. The prevalence of diabetes was 44% and HTN was 33%.

Most patients had tubular coronary lesions (23 patients 51.1%) and 26 patients (57.7%) had single vessel disease.

### Procedural results and in-hospital course

Angiographic and procedural characteristics are summarized in Table 2 and 3.

**Table 2:** Angiographic Characteristics of the 45 Lesions

Characteristics	Mean ± SD
Distal reference vessel diameter (mm)	3.08 ± 0.53
Balloon to artery ratio	1.1 ± 0.3
Maximal balloon inflation pressure (atm)	15.11 ± 2.47
Percent diameter stenosis (%)	81.5 ± 14.5
Angiographic restenosis (%)	4.4
No distal LM lesion n (%)	39 (86.6)
Distal LM lesion n (%)	6 (13.3)
Lesion length (mm)	19.06 ± 8.05

**Table 3:** Clinical Outcomes in the Hospital and During Follow-Up (12 Months)

Clinical events	N (%)
MACE	2 (4.4)
In-hospital	
Procedure success	(100)
Death	0 (0)
Stent thrombosis	0 (0)
Emergency CABG	0 (0)
During follow-up	
Non-related myocardial infarction	2 (4.4)
Death	0 (0)
Target lesion revascularization	2 (4.4)
CABG	1 (2.2)
Target vessel revascularization	0 (0)
Angiographic F/U by symptom of angina pectoris or positive non-invasive test	16 (35.5)
Patients with only clinical F/U	29 (64.4)

Procedural success rate was 100%. Two patients had myocardial infarction in non-related coronary artery during follow-up. The decrease of LCX ostial diameter after stenting was mild in majority of patients, but a moderate narrowing of LCX ostium (< 50%) consistently present. The significant stent jail of LCX was observed at 6 of the 45 lesions (13.3%).

### Angiographic restenosis

Angiographic follow-up data were obtained for 16 of the 45 eligible patients (follow-up rate, 35%), and angiographic restenosis occurred in 2 of 16 patients (12.5%). Two patients were men and had diabetes as risk factor. One patient had total occlusion at proximal to stent and another had focal instent restenosis. First patient referred for CABG and the second patient treated with stenting.

### Late clinical results

**Figure 1:**
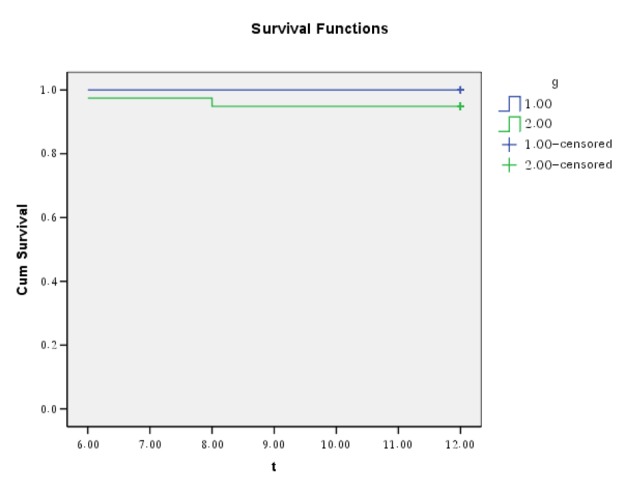
Cumulative analysis of event-free survival was expressed by Kaplan-Meier survival curve. 1: Patients with LM-LAD stenting (n: 6, 13.3%); 2: Patients with focal ostial stenting (n: 39, 86.6%).

Clinical follow-up was available in all patients for one year. There was no death and nonfatal Q wave and non-Q wave myocardial infarction in target vessel during the follow-up. There was no clinically driven TVR or thrombosis during follow-up. Two patients had TLR. Finally event free survival rate (death, myocardial infraction, TLR) was 95.6% at end of the follow-up period (Fig. 1).

## Discussion

The main findings of this study are a high success rate with few complications during hospitalization and follow-up with these new generation stents, these stents are safe without thrombosis and achieve acceptable one-year MACE-free survival.

The use of DES (drug eluting stent) in ostial lesions resulted in more unfavorable outcomes than in non-ostial lesions and previous reports suggested that relatively high restenosis rate (14.7%) might be associated with incomplete lesion coverage [6, 7]. Sirolimus-eluting stents for ostial LAD lesions with full lesion coverage have been shown to reduce restenosis and improve clinical outcomes compared with BMS (bare metal stent) [8]. Several recent studies have documented the safety of LM stenting with DES and early follow-up results did not show excessive morbidity and mortality [9-11]. We used the two stenting strategies like to the technique reported by Seung et al [8]. In our study two patients developed restenosis, one of our patients developed restenosis at the proximal stent and other had focal instent restenosis. That was lower than restenosis rate that reported by Sung et al that evaluated paclitaxel-eluting stents in 2009 [12]. We treated 6 patients with distal LM intermediate (> 50%) lesions and the distal LM segments of stents were usually under higher pressure balloon post dilatation (mean: 18.1 ± 2.0). Cubeddu et al reported that using LM stenting technique for isolated ostial LAD-LCX lesions is a reasonable choice [13]. Park et al showed that stent placement for ostial LAD had 11.7% TLR [14] during two years follow-up and in our study we showed 4.4% TLR during one-year follow-up. The clinical result of our study population regarding MACE-free (95.6%) that was consistent with those obtained in previous studies that have been conducted with DES in ostial LAD lesions [8, 13]. Capranzano et al showed that DES for isolated ostial LAD lesions was a feasible, safe and effective treatment strategy that was similar to our study also they concluded that a default distal LM-LAD stenting, rather than focal ostial stenting, might provide more favorable outcomes [15]. Several registries and a recent subgroup analysis from a randomized trial have shown that DES implantation in LM is a feasible, safe and effective treatment strategy, providing similar results compared to coronary artery bypass grafting, with the only difference being the significantly higher incidence of target vessel reintervention at follow-up after stenting [16-18]. However, the treatment of the LM bifurcation with a single-stent technique, which was the one mostly performed in patients included in the present study, has been shown to provide good results. In agreement with these previous findings, in the present study, given the no TLR among with no reintervention for lesion located at the ostium of LCX, the treatment of LM bifurcation with a single-stent technique using DES appeared to have a good efficacy profile [19, 20]. An interesting observation derived from our study was that two patients that had TLR occurred among patients treated with a stent positioning right at the LAD ostium, although it did not reach the statistical significance. Several factors could have determined this result, among which is the fact that in some patients treated with focal ostial LAD stenting, a distal LM lesion was already present at the time of procedure but was no visible by angiography, and thus, it was left untreated. Therefore, the rationale of extending the stenting of ostial LAD to distal LM is based on the fact that in cases of ostial LAD disease, the plaque frequently extends primarily or secondarily into LM. The fact that showed patient with isolated ostial LAD disease the angiography could fail to show an LM lesion suggested that an IVUS-guided strategy or a default LM stenting coverage may be needed.

This is a single center study and therefore, it has inherent limitations: first, the small number of patients limits statistical power; second, in this study angiographic restenosis rate may be underestimated because in patients that were asymptomatic, have not follow-up angiography and may have restenosis; finally intravascular ultrasound was not used in this study that my improve decision regarding the treatment strategies. However, this is one large study to show stenting of ostial LAD lesion with EES with acceptable angiographic and clinical results.
